# Primary glioblastoma in the pineal region: a case report and review of the literature

**DOI:** 10.1186/1752-1947-2-288

**Published:** 2008-08-27

**Authors:** Kyung-Sub Moon, Shin Jung, Tae-Young Jung, In-Young Kim, Min-Cheol Lee, Kyung-Hwa Lee

**Affiliations:** 1 Department of Neurosurgery, Chonnam National University Research Institute of Medical Sciences, Chonnam National University Hwasun Hospital & Medical School, Gwangju, Republic of Korea; 2 Department of Pathology, Chonnam National University Medical School, Gwangju, Republic of Korea; 3 Department of Pathology, Seonam University, College of Medicine, Namwon, Republic of Korea

## Abstract

**Introduction:**

Glioblastoma in the pineal region is extremely rare with only a few cases reported in the literature.

**Case presentation:**

A 68-year-old man presented with a sudden deterioration manifesting as a headache, vomiting and gait disturbance. A magnetic resonance imaging study revealed a heterogeneously ring-enhanced mass in the pineal region. The mass was subtotally removed through the occipital transtentorial approach, and diagnosed as a glioblastoma.

**Conclusion:**

We discuss the clinical course, radiological findings and treatment strategies of pineal glioblastoma with a review of the relevant literature.

## Introduction

The pineal region consists of the pineal body, the posterior wall of the third ventricle, tela choroidea and velum interpositum. Despite its small size, a wide variety of brain tumors can arise in the pineal region. Tumors of the pineal body may be of pineal parenchymal origin, of extragonadal germ cell origin, or of neuroglial origin [1]. Approximately 11–28% and 50–75% of tumors in the pineal region are pineal parenchymal tumors and germ cell tumors, respectively [1]. In addition, glioma, meningioma and mesenchymal tumors are encountered occasionally. Glioblastoma, which is the most malignant and frequent glioma in brain tumors, is extremely rare in the pineal region with only 17 cases being reported in the literature [2-13]. This paper presents a case of glioblastoma arising in the pineal region and discusses its clinical course, radiological findings and treatment strategies with a review of the relevant literature.

## Case presentation

**Figure 1:**
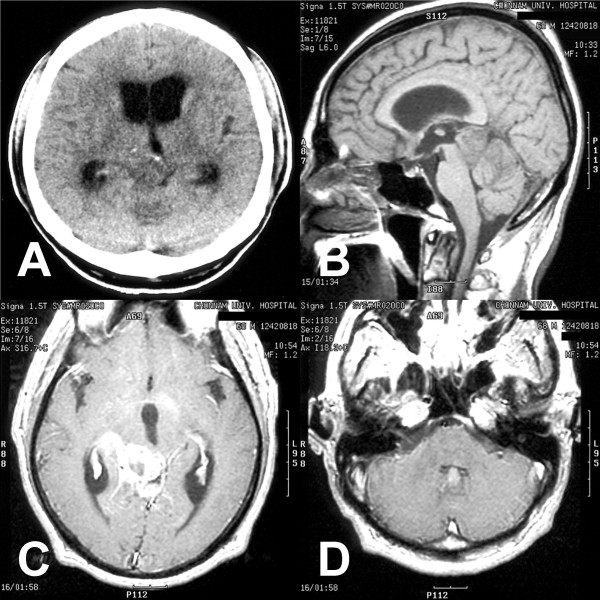
**Non-contrast computed tomography scan showing a hypointense mass in the pineal region (A).** T_1_-weighted sagittal **(B)** and gadolinium-diethylenetriaminepentaacetic acid enhanced axial **(C and D)** magnetic resonance images demonstrating a heterogeneously ring-enhanced mass with central necrosis in the pineal region and ependymal dissemination in the fourth ventricle.

**Figure 2:**
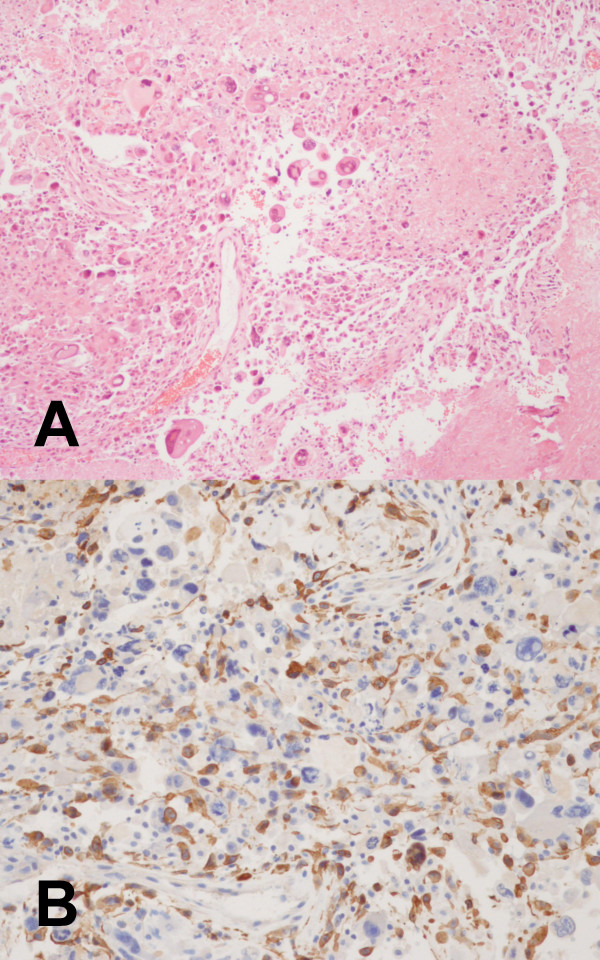
**(A) Photomicrograph showing numerous anaplastic astrocytic tumor cells with mitosis, large multinucleated giant cells with abundant eosinophilic cytoplasm, and an extensive area of necrosis. (B)** Photomicrograph of the immunohistochemical study showing a positive reaction for the glial fibrillary acidic protein (GFAP) (A: hematoxylin and eosin stain, original magnification, ×100, B: original magnification, ×200).

A 68-year-old man presented with a sudden deterioration manifesting as a headache, vomiting and gait disturbance. Two months earlier, he had begun to notice intermittent headaches. Neurological testing revealed ataxic gait features and bilateral papilledema without other neurological deficits. The computed tomography (CT) scan revealed obstructive hydrocephalus caused by a round hypodense ill-defined lesion in the pineal region (Fig. 1A). A magnetic resonance (MR) imaging study demonstrated a 4 × 3 × 4 cm mass at the pineal gland. Through the administration of gadolinium, the lesion showed a heterogeneous hypointensity on the T_1_-weighted image and hyperintensity on the T_2 _image as well as ring-enhancement with an extension into the midbrain and thalamus (Fig. 1B and 1C). No hematological or biochemical abnormalities were evident, and the other tumor markers, such as α-fetoprotein, β-human chorionic gonadotrophin and placental alkaline phosphatase were within normal limits. Surgery was performed using the occipital transtentorial approach because a non-germinomatous malignant tumor was considered a possibility. During the operation, a very soft, gray-colored mass was located in the pineal region, which was barely demarcated from the peritumoral brain. Some hard portions were found in the tumor. An examination of frozen biopsy samples showed anaplastic astrocytic tumor cells. The mass was subtotally removed due to adhesion with the hypothalamus and midbrain, and its severe bleeding nature. The pathologic findings revealed a typical glioblastoma consisting of frequent mitotic figures, a high proliferation index, microvascular proliferation with endothelial cell hyperplasia, and extensive necrosis with focal pseudopalisading (Fig. 2A). Immunohistochemistry revealed a positive reaction to the glial fibrillary acidic protein in both cell bodies and processes (Fig. 2B). A further review of the pre-operative MR imaging study showed an enhanced mass in the fourth ventricle that was consistent with ependymal dissemination (Fig. 1D). Two weeks after surgery, the patient underwent a ventriculoperitoneal shunt due to the rapid exacerbation of signs and symptoms of the hydrocephalus. Considering the pathological and radiological findings, whole neuraxis irradiation therapy was recommended. However, his family insisted on conservative medical support. The patient died 2 months after the diagnosis.

## Discussion

Pineal gliomas include fibrillary astrocytoma, pilocytic astrocytoma, anaplastic astrocytoma, glioblastoma, oligodendroglioma, ependymoma and choroid plexus papilloma [1]. Among these entities, well differentiated astrocytomas are the most common [1]. Since the report of Bradfield and Perez in 1972 [3], only 18 cases including this one have described a glioblastoma of the pineal region (Table 1) [2,4-13]. The patients reported with a pineal glioblastoma consisted of nine women and six men aged from 5 to 68 years (mean, 39.3 years). Compared with those of germ cell or parenchymal tumors in the pineal gland, pineal glioblastomas occur in middle aged adults with a slight female preponderance.

**Table 1:** Summary of reported cases of pineal glioblastoma multiforme

Author/Year	Age/Sex	Symptoms	Radiological findings	Leptomeningeal dissemination	Treatment	Survival
Bradfield et al./1972	53/F	N-A	Obstructive HDC, mass in post. 3rd ventricle	No on autopsy	Resection	Postoperative death
Bradfield et al./1972	5/F	N-A	Obstructive HDC, mass in post. 3rd ventricle	No on autopsy	Shunt	27 mos
DeGirolami et al./1973	3 cases	Intracranial hypertension, vertical gaze palsy in one case	N-A	N-A	RT for all cases, Resection for only one case	N-A
Kalyanaraman/1979	68/F	Ataxia, confusion, urinary incontinence, upgaze limitation	CT: HDC, calcified midline mass	N-A	Resection, RT	4 mos
Norbut et al./1981	36/M	HA, blurry vision, Parinaud's syndrome	CT: HDC, mass in post. 3rd ventricle	Yes on autopsy (4th ventricle, leptomeninges of cerebral cortex, interpeduncular fossa, brain stem, and spinal cord)	Shunt, RT	4 mos
Frank et al./1985	52/F	Intracranial hypertension, oculomotor disturbances	HDC, mass in 3rd ventricle	N-A	Stereotactic biopsy, RT	4 mos
Edwards et al./1988	12/F	N-A	N-A	N-A	Resection, RT, Chemotherapy	18 mos
Vaquero et al./1990	63/M	HA, changing of behavior	CT: rounded hyperdense mass with ring enhancement	N-A	Shunt, Resection, Whole brain RT	6 mos
Pople et al./1993	6/F	HA, N/V, diplopia, decreased visual acuity, 6th cranial nerve palsy, upgaze limitation	CT & MR: HDC, enhancing mass	Yes on FU CT (frontal & occipital lobes, scattered leptomenges)	Shunt, Resection, local RT, Chemotherapy	4 mos
Cho et al./1998	10–15/F	N-A	N-A	N-A	Resection, RT	6 mos
Gasparetto et al./2003	29/F	HA, drowsiness, fever, dizziness, seizure,	CT & MR: ill-defined heterogeneously enhanced mass with extension to thalamus	No	Shunt, Resection	2 mos
Toyooka et al./2005	49/M	HA, diplopia, memory disturbance	MR: irregular heterogeneously enhanced mass	Yes on FU MR (lateral ventricle, pons, pontomedullary junction)	Shunt, Resection, Chemotheraphy (ACNU), local RT	11 mos
Amini et al./2006	40/M	HA, N/V, diplopia, blurry vision	CT: Obstructive HDC, strong enhancement, punctuate calcification MR: heterogenously enhancing with central necrosis, extension into midbrain	Yes on initial MR (cbll, medulla, temporal lobe)	Endoscopic TVB, Resection, Shunt, Whole brain RT, Chemotherapy (Temodar)	5 mos
Amini et al./2006	43/M	HA, disequilibrium, decreased level of mental status	MR: heterogenously enhancing, HDC	Yes on FU MR (intraventricular)	TVB, Resection, Whole brain RT, Chemotherapy	7 mos
Amini et al./2006	52/F	HA, N/V, diplopia, blurry vision, upgaze palsy	MR: heterogenously enhancing with central necrosis, obstructive HDC	Yes on FU MR (lateral ventricle, leptomeninges of brain & spine)	Endoscopic TVB, RT	2 mos
Present case/2006	68/M	HA, N/V, Ataxia	CT: HDC, hypodense mass MR: irregular heterogeneously ring-enhanced mass with central necrosis	Yes on initial MR (4th ventricle)	Resection, Shunt	2 mos

F, female; FU, follow-up; M, male; mos, months; MR, magnetic resonance; CT, computed tomography; HA, headache; N/V, nausea & vomiting; HDC, hydrocephalus; RT, radiation therapy; N-A, not available; post., posterior; TVB, third ventriculostomy & biosy

All reported cases of pineal glioblastomas have presented with signs or symptoms of increased intracranial pressure and hydrocephalus. Eight patients (57.1%) with a pineal glioblastoma also presented with visual or gaze disturbances, including diplopia, blurry vision, nystagmus and upgaze palsy, which were mainly consistent with Parinaud's syndrome. However, the clinical symptoms and signs of pineal glioblastomas are similar to other tumors in the pineal region, which makes them difficult to diagnose based on the clinical history and presentation alone.

MR imaging of pineal glioblastomas demonstrate characteristic features. Heterogeneous enhancement with a centrally located non-enhanced portion indicates central necrosis. Infiltration into the surrounding structures, such as midbrain and thalamus, is shown as hyperintensity on the T_2_-weighted MR image, extending beyond the margin of the enhanced mass. Despite its rapid and infiltrative nature, glioblastomas generally do not invade the subarachnoid space, and rarely metastasize through the cerebrospinal fluid pathway [14]. However, a review of pineal glioblastoma revealed leptomeningeal or ventricular dissemination to be quite common (7 in 10 available cases). Among these cases, two cases, including the present one, showed pre-operative dissemination on the initial radiological study. Upon a careful review of pre-operative MR imaging for a pineal region mass, an enhancing nodule in the subarachnoid space or ventricle system can assist in the diagnosis of glioblastoma.

Considering that most patients with pineal glioblastoma multiforme (GBM) show symptoms and signs of hydrocephalus, an endoscopic third ventriculostomy and tissue biopsy may be an appropriate treatment for pineal glioblastoma. However, according to Amini *et al. *[2], this procedure was unable to resolve the hydrocephalus over time and obtain sufficient tissue samples in two out of three cases. The benefit of an aggressive surgical resection in the treatment of pineal GBM is unclear. Two patients who underwent a surgical resection only, including ours, died 2 months after the diagnosis [8]. The average survival in the three cases who received radiation therapy alone was 3.3 months (range, 2 to 4 months) [2,7,10]. However, adjuvant radiation therapy and/or chemotherapy after a surgical resection may prolong the survival of patients with a pineal glioblastoma. The three patients who underwent a surgical resection and radiation therapy lived an average of 5.3 months (range, 4 to 6 months) [4,9,13]. Furthermore, the mean survival duration of the four patients who received radiation therapy and chemotherapy after the surgical resection was 7 months (range, 4 to 11 months) [2,11,12].

The overall prognosis of a patient with a pineal glioblastoma is poor. Despite every effort in treatment, the maximum survival duration is less than 1 year after diagnosis (except for a single case reported by Bradfield and Perez [3]).

## Conclusion

Glioblastoma in the pineal region is a very rare disease. However, in middle aged patients, a heterogeneously ring-enhanced mass in the pineal region with leptomeningeal dissemination on MR imaging can raise the suspicion of glioblastoma. Even though it is impossible to conclude the best treatment modality, early adjuvant radiation therapy and chemotherapy after surgical resection appear to prolong the survival of patients with a pineal glioblastoma.

## Competing interests

The authors declare that they have no competing interests.

## Authors' contributions

KSM carried out the review of the literature and write up of the manuscript. SJ performed the surgery and was the coordinator of the study. JTY summarized the patient notes and carried out the literature search. KIY participated in the draft of the study, and in the conception of the study. MCL participated in the histopathological analysis, and in the coordination of the study. KHL participated in the draft of the study, and contributed to the work on the histopathology of the case including immunohistochemical work-up. All authors read and approved the final manuscript.

## Consent

Written informed consent was obtained from the patient's relative for publication of this case report and any accompanying images. A copy of the written consent is available for review by the Editor-in-Chief of this journal.
